# Improvement of Ocular Efficacy of Levofloxacin by Bioadhesive Chitosan Coated PLGA Nanoparticles: Box-behnken Design, *In-vitro *Characterization, Antibacterial Evaluation and Scintigraphy Study

**DOI:** 10.22037/ijpr.2019.15318.13016

**Published:** 2020

**Authors:** Ameeduzzafar, Nazia Khan, Nabil K Alruwaili, Syed Nasir Abbas Bukhari, Bader Alsuwayt, Muhammad Afzal, Sana Akhter, Mohd Yasir, Mohammed Elmowafy, Khaled Shalaby, Asgar Ali

**Affiliations:** a *Department of Pharmaceutics, College of Pharmacy, Jouf University, Skaka, P.O. Box 2014, Saudi Arabia. *; b *Department of Pharmaceutics, School of Pharmaceutical Education and Research, Jamia Hamdard, New Delhi, India. *; c *Department of Pharmaceutical Chemistry, College of Pharmacy, Jouf University, Skaka, P.O. Box 2014, Saudi Arabia. *; d *Department of Pharmaceutics, Faculty of Pharmacy, Northern Border University, KSA. *; e *Department of Pharmacology, College of Pharmacy, Jouf University, Skaka, P.O. Box 2014, Saudi Arabia. *; f *Department of Pharmacy, College of Health Science, Arsi University, Asella, Ethopia.*

**Keywords:** Levofloxacin loaded coated PLGA nanoparticles, Box-Behnken, Ocular irritation, Confocal microscopy antimicrobial study, Gamma scintigraphy

## Abstract

Conjunctivitis is considered as a common infection of ocular surfaces. Eye drop is most commonly used for treatment of conjunctivitis, but has some drawback like 95% drug eliminated after administration. Administration of levofloxacin to the anterior site in form of chitosan coated poly (lactic-co-glycolic acid) nanoparticles (LFV-CS-PLGA-NPs) expected to overcome these problem and increasing corneal contact time and permeability for effective treatment of bacterial conjunctivitis. The Nanoparticles were developed by single emulsion solvent evaporation technique and optimized for different variables (chitosan, poly (lactide-co-glycolic acid) and polyvinyl alcohol concentration) by employing three factors, three levels Box–Behnken statistical design. The nanoparticles were evaluated for particle size, drug loading, entrapment efficiency, drug release, *ex-vivo* permeation, ocular tolerance, antimicrobial study, confocal laser microscopy, and Gamma scintigraphy study. The particle size and PdI of the optimized nanoparticles were 169.968 ± 15.23 nm and 0.13 ± 0.03, respectively, where as entrapment efficiency and drug loading is 49.54 ± 2.43% and 11.29 ± 2.13% with extended release profile and strong mucoadhesion. DSC data indicated levofloxacin formed molecular dispersion within coated nanoparticles. Corneal flux showed significantly (*P < *0.05) higher permeation as compared to marketed formulation. Formulation was nonirritant and possessed good antibacterial activity. Gamma Scintigraphy showed slow drainage compared to drug solution, indicating reduction in nasolachrymal drainage. The Gamma Scintigraphy study indicated the CS coated PLGA-NPs have high corneal residence time as compared to drug solution. So, it is revealed that LFV-CS-PLGA-NPs increase the drug concentration over ocular tissue and potential usefulness for sustained drug delivery.

## Introduction

Bacterial conjunctivitis is a common infec-tion referred as pink eye syndrome which is generally caused by numerous bacteria. The common symptoms of bacterial conjunctivitis are lacrimation, hyperemia, irritation, and discharge. Quinolones are employed as first time therapy for conjunctivitis. Levofloxacin is third generation fluoroquinolone used for the treatment of conjunctivitis acts by stopping or preventing the growth of bacteria by inhibiting topoisomerase-IV and DNA gyrase and good activity against *Staphylococcus aureus *on cornea and conjunctiva. Since, levofloxacin is sparingly soluble in water (*i.e. *hydrophobic in nature), it is difficult to dispense the drug in eye drop form due to its poor ocular bioavailability as compared to other water soluble drugs (1). This makes it suitable drug candidate to develop advance drug delivery (prolonged delivery) system with reduced dosing frequency and side effects.

Topical route is the most convenient route in ocular drug delivery. The low corneal residence time, poor bioavailability is frequently observed in conventional topical eye drops. Only 5% of the drug remains for biological activity and 95% drug eliminated through nasolacrimal drainage, blinking and dilution with tear fluid with conventional eye drop (2-3). Due to these limiting factors, researchers made many efforts to enhanced the corneal contact time and bioavailability via different types of novel formulations like *in-situ* gel system (3-5), and nanoparticulate system (6) using different types of bioadhesive and biodegradable polymers. 

Among all formulation, polymeric nanoparticles are of prime importance in ocular drug delivery. Enhance in corneal and conjunctival absorption of drugs to attain at desired levels and reduction in systemic side effects by decreasing the nasolacrimal drainage (6-8) is possible with polymeric nanoparticles. Various natural as well as synthetic biodegradable polymers like chitosan (CS) (9), alginate (10), pectin, poly lactic-co-glycolic acid (PLGA) (11-12) and polylactide acid (PLA) (13) are exceedingly employed for the development of polymeric nanoparticles.

Among synthetic biodegradable polymers used in fabrication of nanoparticles, PLGA, a copolymer of poly lactic acid (PLA) and poly glycolic acid (PGA), is the best biomaterial used for conveyance of sparingly soluble drugs till now. These polymers are relatively safe for human use due to its biodegradable nature and employed as a raw material for nanoparticle production (11, 14-16).

Recently, mucoadhesive ocular drug delivery has been investigated for topical route of drug administration (17). Chitosan, hydrophilic polysaccharide with cationic properties, is extensively studied for ophthalmic drug delivery (18). The strong binding property (bioadhesive) of chitosan is due to binding with negatively charged mucin layer of conjunctiva and corneal surface. Bioadhesive nanoparticle is an alternative approach for improving the bioavailability of drug to ocular tissue (19). Further the antibacterial activity of chitosan helps to lessen the post-infection after ocular drug administration (20).

Entrapment of hydrophobic drug in nanoparticulate system using lipophilic polymer and coating by bioadhesive polymer (chitosan) provides a good approach for ocular drug retention, transcorneal permeation and improves in ocular bioavailability of drug. 

## Experimental

Poly (lactic-co-glycolic acid) copolymer (PLGA, ratio M/M%: 50/50; 33 kDa) was procured as gift sample from Purac Biochem BV (Gorinchem, the Netherlands). Chitosan (CS, MW: 120,000, with a deacetylation degree >80%) was obtained from India Sea Foods (Kochi, Kerala, India) as a gift sample. Polyvinyl alcohol (PVA; MW 95,000) was acquired from Sigma-Aldrich (St. Louis, MO). Levofloxacin was procured from Unicure Pvt. Limited, Noida, UP India as a gift sample. Milli-Q water from water purification system (Millipore, Billerica, MA) in the research facility was utilized in this investigation. All other chemicals and reagents used were of analytical grade and procured from Sigma-Aldrich (St. Louis, Missouri, USA.).

New Zealand albino rabbits either sex weighing (1.8–2.2 kg) were selected for study of scintigraphy. The rabbits were kept in the animal house. The food and water were supplied as per guidelines approved by the Institutional Animal Ethics Committee (IAEC), Jamia Hamdard. The study protocol was approved by the Institutional Animal Ethics Committee (IAEC), Jamia Hamdard and conducted under the Committee for the Purpose of Control and Supervision of Experiments on Animals (CPCSEA) guidelines at Institute of Nuclear Medicine and Allied Sciences (INMAS), Delhi, India.

*High performance liquid chromatography (HPLC)*

The High performance liquid chromatography (HPLC) linked with UV-detector was used for quantification of drug content (Shimadzu, Model LC-10 ATVP) Japan). The HPLC was equipped with a binary pump and UV detection system (SPD-10A). The C_18_ column (average particle size 5 μm, 250 mm × 4.6 mm) was used for chromatographic separation. The mobile phase consisted of acetic acid and acetonitrile in the ratio of 80:20 (v/v) at a flow rate of 0.5 mL/min. The injection volume was 20 µL for standard and samples. The standard and samples were filtered through 0.45 µm filter.

*Preparation of levofloxacin loaded chitosan coated PLGA nanoplex*

Levofloxacin loaded CS-coated PLGA-NPs (LFV-CS-PLGA-NPs) were prepared by single emulsification (o/w) solvent evaporation technique (21). Different concentrations of lipophilic polymer (PLGA, 0.2-0.6%) and levofloxacin were dissolved in organic solvent (dichloromethane DCM, 2mL) initially. Organic phase solution was dropped in aqueous solution containing different concentrations of CS (0.2 to 1%) and PVA solution (prepared in 1% acetic acid solution) on magnetic stirrer (1200 rpm, REMI, Mumbai, India).The o/w emulsion was formed and continuously stirred for 8 h at room temperature for complete evaporation of organic solvent (DCM, limit no more than 600 ppm) (22). The formulation was centrifuged at 18,000 rpm at 4 ºC (REMI Cooling Centrifuge, Mumbai, India), and washed two times with Milli-Q water. The separated nanoparticles were resuspended in 2 mL of Milli-Q water and further dried in a lyophilizer (Heto DRYWINNER, Germany) using mannitol (1% w/v) as cryoprotectant.

*Optimization by Box-Behnken statistical design*

Three factors and three levels Box-Behnken statistical design were used for the optimization of CS-coated PLGA-NP’s of levofloxacin because it gives less number of experiments and save more amount of drug as compared to the others techniques. Concentration of PVA in aqueous phase (%, A), concentration of PLGA in organic phase (%, B), and concentration of CS in aqueous phase (%, C) were selected as independent variable whereas particle size (Y_1_), entrapment efficiency (Y_2_) and drug loading (Y_3_) were selected as dependent variables (Table 1). The independent variables range was selected according to the results of preliminary screening (data not showed). The Quadratic model obtained using Design-Expert 8.0.7.1 software (Stat-Ease Inc., Minneapolis, MN). Total 17 experimental runs were obtained from the software, and prepared in triplicate (Table 2). The mathematical relationship of dependent variables (Y_1_, Y_2_, and Y_3_) with independent variables (A, B and C) was developed by polynomial equation as follows.

Y= β_0_ + β_1_A + β_2_B + β_3_C + β_12_AB + β_13_AC + β_23_BC+ β_11_A^2 ^+ β_22_B^2 ^+ β_33_C^2 ^+ …

Where, Y denotes predicted response(s); β_0_ is the intercept; and β_1_, β_2_, and β_3_, denotes linear coefficients. The β_11_, β_22_, and β_33_, represent squared coefficients and β_12_, β_13_, and β_23_, the interaction coefficients of the equation. The best fit model was obtained by analysis of variance (ANOVA). The statistical significance of the data was performed in terms of regression coefficients.

The relationship of the dependent to independent variables was evaluated using contour and three-dimensional (3D) response graph. Optimum concentrations of independent and dependent variables were selected from point prediction and compared with the experimental value to validate the chosen experimental domain and polynomial Equation (Table 3). 

*Characterization of nanoparticles*

*Particle size and polydispersibility index (PdI)*

The particle size and PDI of CS coated PLGA-NPs determined by dynamic light scattering method using zeta sizer instrument (Zetasizer Nano Range, Malvern instruments, Malvern, UK). The zetaziser was operated at accelerating voltage (13.52 kV) under high vacuum.

*Transmission electron microscopy*

Morphology and exact particle size of the NPs were determined using transmission electron Microscopy (TEM, Philips CM 10, Eindhoven, City, Holland). CS coated PLGA-NPs suspension (2–10 µL) was dropped on a 300 mesh copper grid (carbon coated film), and stand for 10 min for air dried. For staining the samples, 2% w/v phosphotungstic acid solution was employed for 2 min and excess volume was soaked with filter paper and subsequently placed in transmission electron microscopy. Soft Imaging Viewer software was used for image capture and analysis.

*Differential scanning calorimetry (DSC) analysis*

The automated DSC system (Perkin Elmer Pyres 6 DSC, USA) was used for DSC analysis of sample. Approx 3-5 mg of all the samples were kept in DSC pan, sealed, and analysed under steady nitrogen purging atmosphere at 30 °C to 350 °C at the rate 10 °C min^-1^.

*Determination of drug encapsulation efficiency (EE) and drug loading (DL)*

The EE of NPs was measured by centrifugation technique. The nanosuspension was centrifuge using cooling centrifuge (18000 rpm, 4 ºC, 10 min, C24, REMI Cooling Centrifuge, Mumbai, India). The supernatant was collected and analyzed by HPLC. The percentage EE and DL was calculated by given Equations 1 and 2.

$\mathrm{EE\%}=\frac{\mathrm{Total amunt of drug}-\mathrm{Amount of drug in supernatant}}{\mathrm{Total amunt of drug}}\times 10$          (Equation 1)

$\mathrm{DL\%}=\frac{\mathrm{Totalamuntofdrug}-\mathrm{Amountofdruginsupernatant}}{\mathrm{Weightofnanoparticles}}\times 100$          (Equation 2)

*In-vitro drug release study*

Dialysis bag method was used for *in-vitro *drug release study of NPs. Drug loaded CS-PLGA-NPs suspension (2 mg equivalent drug in 200 µL buffer) was placed in dialysis bag (cellulose membrane; molecular weight cut-off: 12 kDa). The dialysis bag pre-soaked with distilled water for 24 h, then used for study. Ten milliliter of dissolution media (Dulbecco’s phosphate-buffered saline (DPBS) can be used to provide a buffer, Glucose, NaCl, KCl, Na_2_HPO_4_, KH_2_PO_4_, CaCl_2_, MgCl_2_, pH 7.4, mimic to tear fluid) at 37 ± 2 ºC under continuous magnetic sterring (150 rpm). At definite time intervals (1, 2, 3, 4, 5, 6, 8, 10, 12, and 24 h) 1 mL sample was withdrawn and the same volume was replaced with pre-equilibrated 37 ± 2 ºC fresh buffer medium. The drug content was quantified by HPLC with UV detector at 288 nm. The release graph was plotted time *vs.* cumulative drug release. The drug release profile was fitted into various release kinetic models like zero order, First order, Higuchi’s model, and Korsmeyer-Peppas for determination of the best model.

*Transcorneal permeation study*

The transcorneal permeation study was done using Franz diffusion cell on goat eye cornea. The goat eye was collected from a local slaughter house and preserved in normal saline at 4 °C. The cornea was removed carefully with approx 1 mm sclera from all sides. The excised cornea (exposed surface area 0.64 cm^2^) was placed between receptor and donor compartment of Franz diffusion cell. The LFV-CS-PLGA-NPs-Opt suspension (200 µL) was kept in the donor compartment. The Dulbecco’s phosphate-buffered saline (Glucose, NaCl, KCl, Na_2_HPO_4_, KH_2_PO_4_, CaCl_2_, MgCl_2_, pH 7.4, mimic to tear fluid) was used as dissolution media kept in the receptor compartment at 37 ± 2 ºC under magnetic stirring (150 rpm). One milliliter of the sample was withdrawn at predetermined time interval (0, 2, 6, 8, 10, 12, and 24 h) from the receptor compartment of diffusion cell and the same volume was replaced with Dulbecco’s phosphate-buffered saline. The amount of the drug permeated was determined by HPLC and compared with marketed eye drop (Levobact, 0.5%).

*Ex-vivo mucoadhesion study*

The *ex-vivo *mucoadhesion of LFV-CS-PLGA-NPs-Opt was studied using pig mucin (Himedia, Mumbai, India) because it is similar with corneal mucin (23). The nanoparticle suspensions (placebo and drug loaded NPs) was incubated with 1% pig mucin suspension (PBS, 0.05 M, pH 7.4) at 37 ºC for 24 h (9) and centrifuged at 18000 rpm for 30 min at 4 ºC (REMI Cooling Centrifuge, Mumbai, India), the supernatant was collected and evaluated for free pig mucin using UV spectrophotometer (Shimadzu UV-1700, Kyoto, Japan) at 280.2 nm. The mucoadhesion strength was calculated using equation.

Bioadhision%=Initial conc of pig mucin-free conc of pig mucin Initial conc of pig mucin×100

*Ocular tolerance study: Hen’s Egg Test Chorioallantoic Membrane (HET- CAM) test*

The *in-vitro* ocular tolerance test by HET-CAM is an alternative method to Draize Rabbit Eye Test (24). HET-CAM has been employed extensively to check the ocular irritancy of any ophthalmic product. The fertilized fresh hen’s eggs (50-60 g) was collected from local poultry form and divided into three groups, each containing three eggs. The eggs were incubated in a humidified shaking incubator at 37 ± 0.5 ºC and 55 ± 7% RH for 9 days and rotated manually after every 12 h. On 10^th^ day of incubation the shell of egg was open from air chamber side and the inner membrane removed without any damage to vascular CAM. The LFV-CS-PLGA-NPs-Opt suspension, normal saline (0.9%, negative control), and sodium hydroxide (0.1 M, positive control) were applied directly to the CAM surface and stand for 5 min without any turbulence. The CAM membrane was inspected for any sign of vascular damage such as hyperemia, hemorrhage, and coagulation and scored (0 to 21) according to the scoring scheme. At predetermined time intervals (0, 0.5, 2, and 5 min) the CAM was observed and the scores were given according to the nature of hemorrhage and the mean was calculated. No visible hemorrhage = 0 Nonirritant. Just visible membrane discoloration = 1 Mild irritant. The structures are covered partially due to membrane discoloration or hemorrhage = 2 moderately irritant. The structures are covered totally due to membrane discoloration or hemorrhage = 2 Severe irritant (25). 

*Histopathological study*

Corneal irritation study of LEV-CS-PLGA-NPs-Opt formulation was analyzed by using fresh goat cornea. The cornea was incubated with LEV-CS-PLGA-NPs-Opt for a definite e period of time. The cornea was washed with phosphate buffer saline, and placed fixed in formalin (8% v/v) solution. Cornea was dehydrated with alcohol, put in melted paraffin and solidified. Definite cross-section was cut and stained using haematoxylin and eosin. Snaps were captured by Motic digital (DMB3, Pal System, Japan) microscope at 10x and evaluated for any change in corneal anatomy and compared to the controlled cornea.

*Confocal laser scanning microscopy study (CLSM)*

Confocal scanning laser microscopy study was done to determine penetration of formulation in excised goat cornea. The fluorescent dye (0.3%, Rhodamine B) was used for study. The excised goat cornea was obtained from the local slaughter house in normal saline. The cornea was placed with LEV-CS-PLGA-NPs-Opt formulation, dye in Franz diffusion shell for 8 h, and maintained at 37 ± 2 ºC. The goat cornea was separated, washed, and cut into the small pieces. The microscopy slide was prepared. The confocal scanning laser microscopy (Olympus FluoView FV1000, Melville, New York) with an argon laser beam was used for study with excitation and emission wavelength 370 nm and 550 nm. The image was captured by z-axis by FluoView software at Z-axis (7, 11).

*Antimicrobial study*

The antibacterial activity of LEV-CS-PLGA-NPs-Opt and marketed eye drops (levobact, 0.5%) was carried out against *Staphylococcocus aureus *(*S. aureus*) microorganism. The sterilized nutrient agar media (20 mL) was placed in sterilized petriplate and seeded with specific culture (0.2 mL). The petriplate was allowed to stand for solidification without any disturbance. Four millimeter diameter cups were made with the help of sterile borer. The 50 mg of LEV-CS-PLGA-NPs-Opt were taken and resuspended in normal saline. After 12 and 24 h the released levofloxacin from NPs transferred into the cups for microbial assay and compared with marketed eye drops, (Levobact, 0.5%). With another petridish. The petridishes kept at room temperature for 3h, and incubated at 37 °C for 24 h. Diameter of zone of inhibition (ZOI) was determined by antibiotic zone finder.

*Gamma scintigraphy: Ocular retention study*

The ocular retention of developed optimized formulation was accessed by Gamma Scintigraphy using Tc^99m^ as radioactive substance. Initially levofloxacin is direct radiolabelled with dry Tc^99m^ using stannous chloride as reducing agent. The rediolabelling efficiency was determined with acetone as mobile phase using instant thin layer chromatography (ITLC) and optimized. Radiolabelled levofloxacin used to develop CS coated PLGA-NPs with optimized formula. The study was performed on Gamma Camera (Millenium VG, Milwaukee, WI); to detect the 140 keV radiation of Tc^99m^. The rabbit was anaesthetizing intramuscularly using Ketamine HCl injection (15 mg/kg body weight). The 50 µL of the radiolabelled levofloxacin loaded CS coated PLGA-NPs suspension and aqueous levofloxacin solution (50 µL) were instilled on the left side corneal cul-de sac. Scintigraphy dynamic recording continued for 30min with dynamic images that were captured with high resolution camera in scintigraphy (128 × 128 pixel). The drainage rate of radioactive compound from eye was measured and the time- activity curve plotted. The static image of whole body was captured (128 × 128 pixel) at definite time interval up to 6 h after instillation of both formulations (NPs and drug solution) (8).

*Stability study as per ICH-Guideline*

The stability study of the freeze dried LEV-CS-PLGA-NPs-Opt were done as per ICH guide line (26). The 40 mg of freeze dried CS-coated PLGA-NPs were packed in closed amber colored glass vials and placed at 4, 25, and 40 °C in humidity chamber. The 10mg of the LEV-CS-PLGA-NPs-Opt was withdrawn at definite time interval (1, 2, 3, and 6 month) and particle size, encapsulation efficiency, drug loading, and drug content was analyzed. HPLC method was used for determination of drug concentration with mobile phase methanol: water (70:30, v/v) at UV detector 288 nm. The degradation rate constant and self life of formulation was calculated by following Equations 3 and 4. Graph pad prizme was used for statistical analysis. The *P < *0.05 means statistically significant and *P *> 0.05 means statistically insignificant.

Slope = K/2.303                    (Equation 3)

Self life t_90_ = 0.1052/K_25 _                     (Equation 4)

## Results and Discussion

*Optimization*

**Figure 1:**
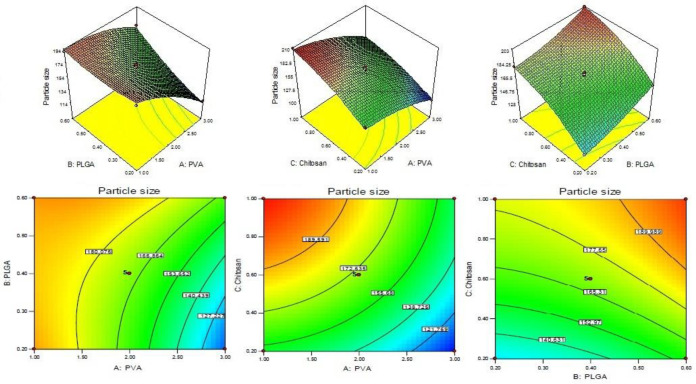
3D and couture plot showing effect of (A) PVA, (B) PLGA and (C) Chitosan on response Y_1_ (particle size).

**Figure 2.**
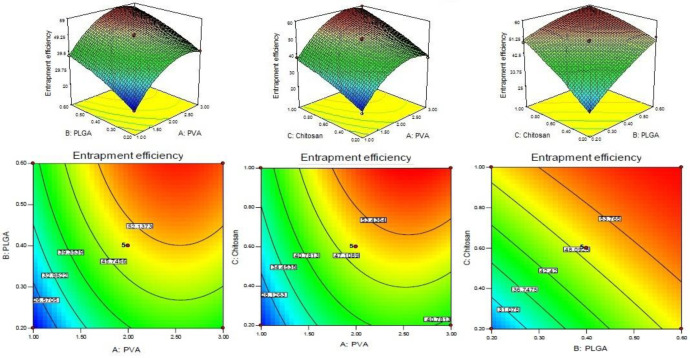
3D and couture plot showing effect of independent variables *i.e.*, (A) PVA, (B), PLGA, and (C) Chitosan on response Y_2_ (entrapment efficiency)

**Figure 3:**
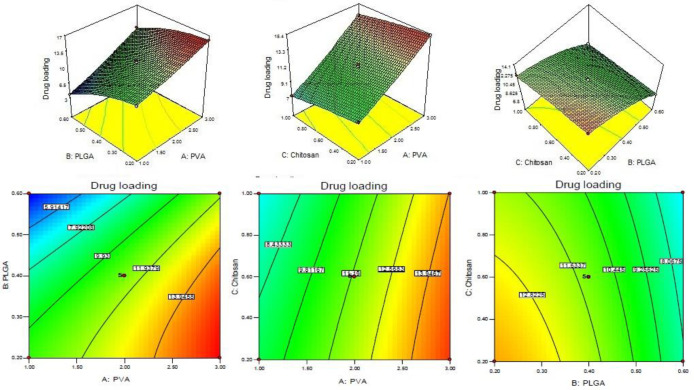
3D and couture plot showing effect of independent variables *i.e.*, (A) PVA, (B) PLGA, and (C) Chitosan on response Y_3 _on drug loading

CS coated PLGA-NPs of Levofloxacin was developed and optimized by Box-Behnken statistical design using Design-Expert software. Total 17 runs with five centre points and their responses were recorded (Table 2). The best fit model for each response *i.e.*, particle size, entrapment efficiency, and drug loading were selected by ANOVA and by calculating *F*-values. A full-quadratic second-order polynomial equation was generated for each response (particle size, entrapment efficiency and drug loading). The ANOVA and regression analysis of each model were shown in Tables 3 and 4, respectively. The 3D and contour plots provided utility in analyzing the effects of two factors on one particular response and helped to demonstrate the interaction effects of the independent variables on the dependant variables (response) shown in Figures 1-3.

*Particle size*

The particle size of every batch of CS coated PLGA-NPs was measured by zeta sizer and put in to the Design-Expert software. The quadratic polynomial Equation was given bellow.

Particle size (Y_1_) = +169.97 - 26.65A + 12.80B + 24.21C + 12.05AB - 2.98AC - 1.19BC - 9.12A^2 ^+ 4.67B^2 ^- 8.14C^2^. 

From the result of ANOVA, the Model F-value of response Y_1 _(particle size) is 102.64, indicating that the model is significant. The model terms are considered significant with values of “Prob > *F*” less than 0.0500. In this case the significant model terms are A, B, C, AB, A^2^, B^2^, and C^2^. The insignificant model terms are indicated with values greater than 0.1000. The concentration of CS varied from 0.2 to 1% w/v. The particle size of NPs varies from 102.60 ± 4.34 to 208.76 ± 6.34 nm (Table 2). With increase in concentration of CS, a gradual increase in particle size was observed due to increase viscosity of solution (Figure 1). A significant (*P < *0.05) increase in particle size was observed when CS concentration increased from 0.2% to 0.6% w/v (*P < *0.05). With increased concentration of PLGA, the particle size increased due to increase in the viscosity of solution resulting in increase in resistance against force created by stirring (Figure 1) (27). In case of stabilizer or emulsifier *i.e.*, PVA, on increasing the concentration of emulsifier in the external phase (1-2%), the particle size decreases significantly (*P < *0.05, Table 2, Figure 1) because at this concentration, the PVA molecule acts as a stabilizer and prevents aggregation of the particles. Above 2%, the particle size increased due to increase in viscosity, resulting in decrease in share force for breaking the particles.

*Entrapment efficiency*

The entrapment efficiency of each batches of LFV-CS-PLGA-NPs determined by centrifugation technique and value was inter in the Design-Expert software. The quadratic polynomial Equation is given below.

Entrapment efficiency (Y_2_) **= **+49.45 + 9.71A + 8.49B + 8.53C - 0.23AB + 0.86AC - 3.76BC - 8.92A^2 ^- 1.93B^2 ^- 1.35C^2^

The model quadratic model was found to be significant with *F*-value of 144.65. The significance of the model terms is indicated by values of “Prob > *F*” less than 0.0500. The A, B, C, BC, A^2^, and B^2 ^are significant model terms and remaining is insignificant because the *P *> 0.1. If there are many insignificant model terms (not counting those required to support hierarchy), model reduction may improve your model. Significant Lack of Fit value is implied with a “Lack of Fit *F*-value” of 51.72. Due to noise a 0.12% chance is that a “Lack of Fit *F*-value” this large could occur. The EE of levofloxacin increases on increasing the concentration of PVA because it forms a uniform and stable dispersion which prevents loss of drug from coated nanoparticles (*P < *0.05, Figure 2). On increasing the PLGA content from 2 mg/mL to 6mg/mL the EE significantly increased (Figure 2) due to less diffusion of the drug from the NPs. From the polynomial equation, a direct positive relationship with CS concentration was observed which means that with increase in the concentration of CS, EE increased because of high viscosity and higher extent of encapsulation and lesser diffusion of drug from NPs (Figure 2) (9, 28).

*Drug loading*

The drug loading of levofloxacin in CS coated PLGA-NPs of all batches was determined by centrifugation technique and data was given in Table 2. The polynomial Equation from ANOVA was calculated which is given below. 

Drug loading (Y_3_) = +11.29 + 3.30A - 2.73B - 0.84C + 0.62AB + 0.18AC + 0.18BC + 0.18A^2 ^- 0.93B^2 ^- 0.11C^2^

Assessment of a particular factor effect on a response is generally represented by a positive or negative value. A favourable effect is indicated by the positive value for the coefficient whereas an unfavorable effect is indicated by negative value respectively.

The model *F*-value was found to be 754.55, which implied that the model is significant. The A, B, C, AB, BC, A^2^, and B^2^ was found to be significant model term because the *P < *0.05. The predicted R^2^ of 0.9863 is in reasonable agreement with the adjusted R^2 ^of 0.9976. The adequate precision was found to be >4 (103.098) indicated that the method has adequate signal. The drug loading for all batches is given in Table 2. From the polynomial Equation the PVA showed direct positive relationship with DL whereas PLGA and CS have negative relationship with DL. With increases in PVA concentration, the DL increased because the particle size decreased due to prevention of particle agglomeration., The DL was decreases on increasing the PLGA and CS concentration because of high viscosity of solution and large particle size formation (28) (Figure 3). The LFV-CS-PLGA-NPs-Opt formulation has concentrations of PVA (2%): PLGA (0.5%): CS (0.6%) has a particle size of 169.968 ± 15.23 nm, entrapment efficiency of 49.54 ± 2.43% and drug loading of 11.29 ± 2.13%.

*Particle size and Transmission electron microscopy (TEM)*

**Figure 4:**
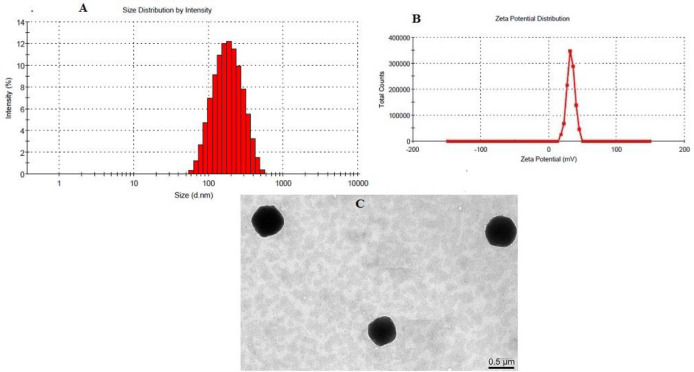
(A) Particle size, (B) Zeta potential and (C) TEM photograph of optimized chitosan coated PLGA-NPs

The optimized formulation had a particle size of 169.968 ± 15.23 nm with PdI (0.13 ± 0.03) as shown in Figure 4A. The CS coated PLGA-NPs had positive zeta potential as expected due to the positive charge of the CS polymer. 28.5 mV (Figure 4B) was the zeta potential of the optimized formulation which confirms CS coating over PLGA-NPs. TEM image of LFV-CS-PLGA-NPs-Opt was determined and found to be spherical shaped as well as defined core shell structure of NPs (Figure 4C).

*Differential scanning calorimetry*

**Figure 5:**
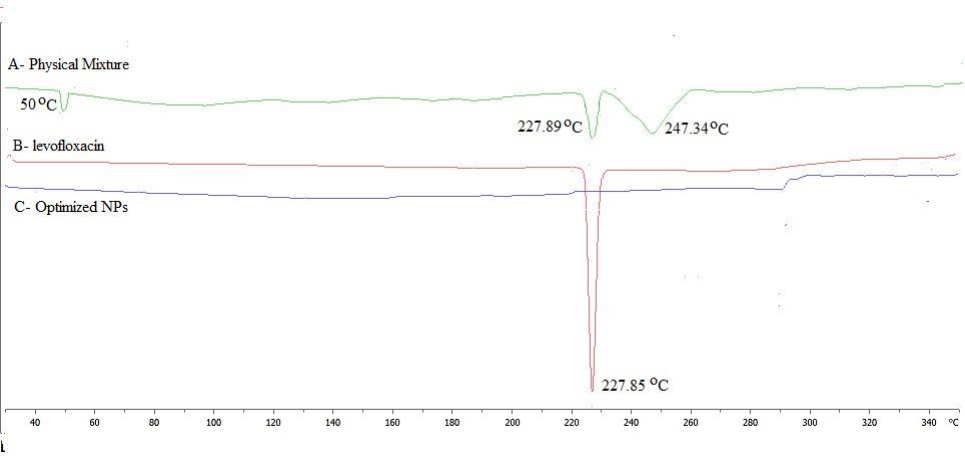
DSC of (A) Physical mixture, (B) Levofloxacin, (C) Optimized NPs

The DSC thermogram of levofloxacin, physical mixture (levofloxacin with polymers) and LFV-CS-PLGA-NPs-Opt were performed and shown in Figures 5A-5C. The thermogram of levofloxacin, in pure drug and in physical mixture showed characteristic intense endothermic peak at 227.85 ºC (Figures 5A and 5B) at its melting point confirmed that its crystalline in nature The LFV-CS-PLGA-NPs-Opt did not show any characteristics peak (Figure 5C), indicating that levofloxacin was completely encapsulated by the polymer matrix and also due to nanosizing (29-32).

*In-vitro release study*

**Figure 6:**
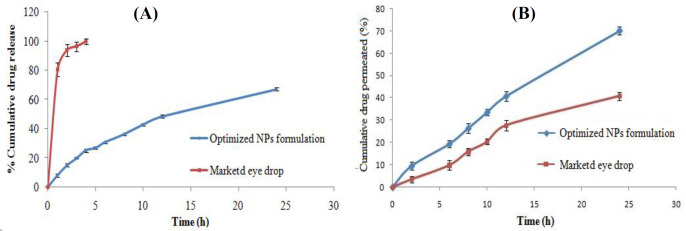
(A) *In-vitro* drug release profile, (B) *ex-vivo* permeation study

The *in-vitro *release of levofloxacin from LFV-CS-PLGA-NPs-Opt and marketed formulation (levobact, 0.5%) were performed (Figure 6A). Slow but consistent drug release was observed. The LFV-CS-PLGA-NPs-Opt showed 8.24 ± 1.137% released in 1 h followed by 67.21 ± 1.224% in 24 h, while the marketed formulation exhibited 80.65 ± 4.54% release in 1 h followed by 99.97 ± 1.87% release 4 h. The LFV-CS-PLGA-NPs-Opt formulation showed slow and sustained release of drug as compared to marketed formulation (Figure 6A) due to the additional layer of CS present on the PLGA-NPs surface which acts as supplementary barrier to the drug diffusion (33), that means the drug molecule takes longer time in diffusion of drug in to release medium from polymer core and this result agrees with the earlier published results (34). The release profile of LFV-CS-PLGA-NPs-Opt was fitted into different kinetic release models and higuchi model showed best release model with regression value (R^2^) of 0.982 (Table 5). It indicated drug release from nanoparticle matrix by diffusion process which is based on Ficks law and square root of time dependent (35).

*Transcorneal permeation study*

The corneal permeation of LFV-CS-PLGA-NPs-Opt and marketed formulation was assessed through diffusion cell. The percentage of drug permeation and flux of levofloxacin from the LFV-CS-PLGA-NPs-Opt found to be significantly (*P < *0.05) high (70.206%, 2.90 ± 0.036 µg/cm^2^/h) as compared to marketed formulation (40.764%, 1.776 ± 0.067 µg/cm^2^/h). The flux was determined from the steady-state portion of the permeation graph (cumulative amount permeated per unit area verses time (Figure 6B). The Higher corneal permeation was observed with LFV-CS-PLGA-NPs-Opt as compared to marketed formulation due to permeation enhancing activity of CS as well as the nano size of coated NPs. Another reason for the enhanced permeation might be positively charged CS (positively charged due to presence of amino groups) interaction with negatively charged corneal surface (7).

*Ex-vivo mucoadhesion study*

The *ex-vivo* mucoadhesion of for LFV-CS-PLGA-NPs-Opt was determined using pig mucin and found to be 94.15 ± 1.72%. This mucoadhesion is due to hydrogen bond between positively charged amino group of CS and negatively charged mucin (oligosaccharide chains) (36).

*Ocular tolerability test (HET-CAM Test)*

**Figure 7:**
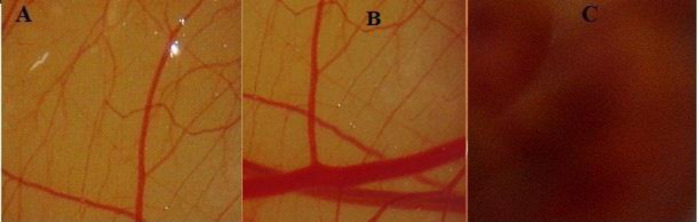
HET-CAM image of treated with (A) NPs (B) Negative control (NaCl) (C) Positive control (0.1N NaOH).

Ocular irritation study of the developed LFV-CS-PLGA-NPs-Opt was assessed by HET-CAM test. HET-CAM is considered as a best choice and a suitable method for assessment of irritancy as compared to the animal testing (Draize test). Ethical and legal obligations are not conflicted by this test. Quite similar to the inflammation induced in the rabbits eye conjunctival tissue during irritancy studies, this test also responds to the injury inflicted with a complete inflammatory process (37). The optimized formulation tested and results were compared with non-irritant normal saline (0.9% NaCl, negative control). A zero mean score was found for normal saline. The LFV-CS-PLGA-NPs-Opt showed 0 score up to 12 h and 0.46 score up to 24 h indicating that NPs formulation is non irritant due to nanorange of particles as well as due to isotonicity of formulation (38). In case of positive control (NaOH), the mean score was fond to be 14.33 which clearly confirmed its irritant nature. Hence, the results clarified that the LFV-CS-PLGA-NPs-Opt was non-irritant and can be well tolerated (Figure 7).

*Histopathological study*

**Figure 8:**
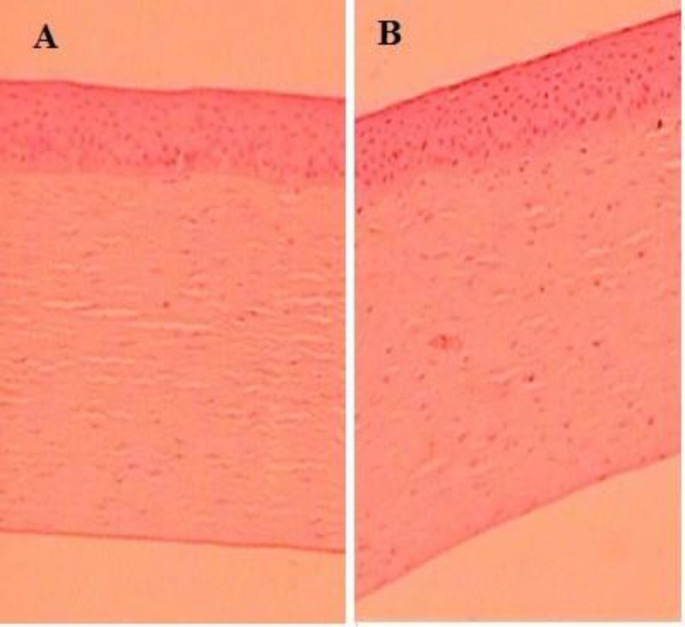
Histopathological image (A) treated with NPs, (B) Normal control

Figure 8 represent the Histopathological study of LFV-CS-PLGA-NPs-Opt and with negative control (0.9% w/v, NaCl) and that and it was found that there was no change in morphological structure of ocular layer. 

*Confocal laser scanning microscopy study*

**Figure 9:**
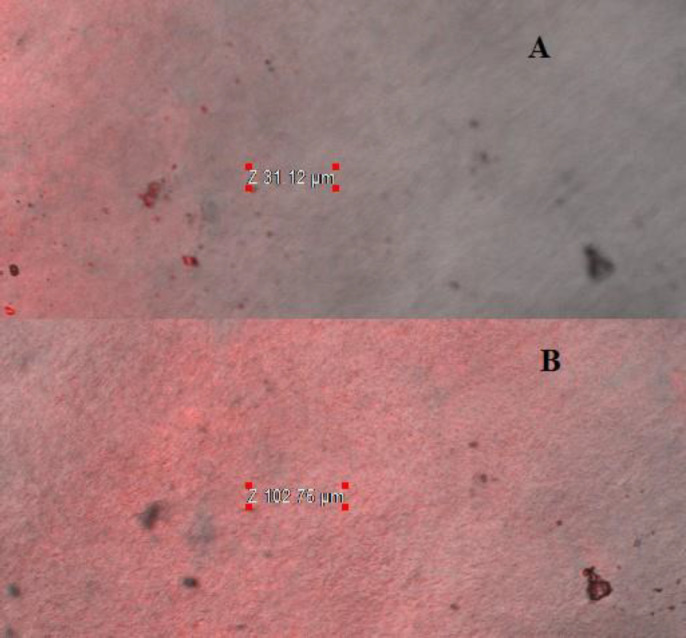
Confocal scanning laser microscopy of (A) optimized CS coated PLGA-NPs, (B) marketed eye drop

The CLSM was performed on excised goat cornea using fluorescent dye (0.3% Rhodamine dye) to determine the extent of penetration of NPs and drug solution. It was observed that LFV-CS-PLGA-NPs-Opt showed high penetration (102 µm) with high florescent intensity as compared with marketed preparation (31.12 µm) (Figures 9A and 9B). The deep penetration of NPs is due to binding of positively charge on NPs to negative charge of cornea and tight junction. It loosen the tight junctions of epithelial cell (change the relative concentrations of specific ion species in the pore volume) and allow transport of NPs paracellularly (39). The long residence time on cornea is not only by simple bio-adhesion, but also by transport of NPs through intercellular as well as intracellular pathway through cornea. It concluded that the CS-PLGA-NPs-Opt penetrated deeper corneal layer on only adsorbed on corneal surface. This result is agreed with the previous published research work (7).

*Antimicrobial study*

The antibacterial efficacy of LFV-CS-PLGA-NPs-Opt with marketed eyed drop was performed against* S. aureus*. The diameter of ZOI was measured by graduated scale. The ZOI of marketed eyed drop was 25.12 ± 0.65 mm as compared to LFV-CS-PLGA-NPs-Opt (30.74 ± 0.25 mm at 24 h). The results exposed that developed LFV-CS-PLGA-NPs-Opt is significant (*P < *0.05) higher antibacterial activity than marketed formulation.

*Ocular retention: scintigraphy study*

**Figure 10.**
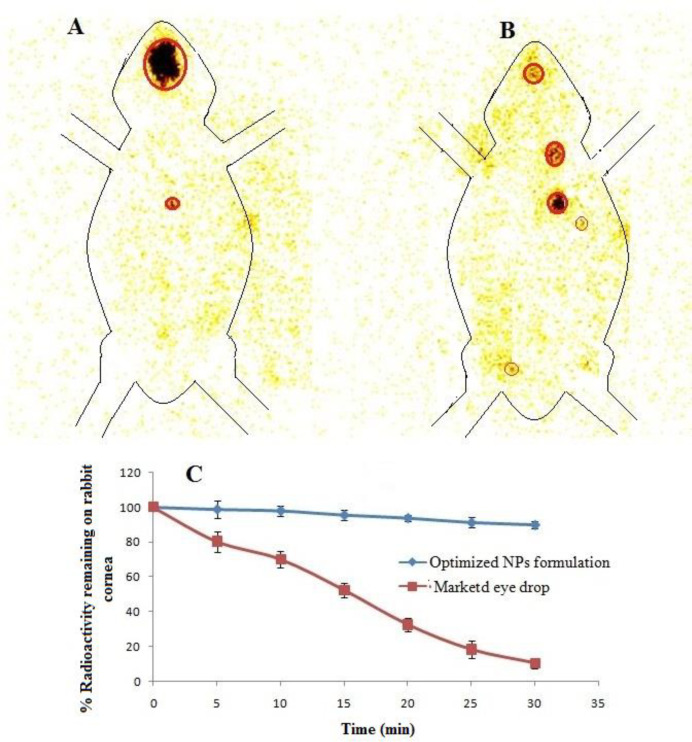
(A) Scintigraphy static image for CS coated PLGA-NPs, (B) marled eye drop, (C) time activity plot for CS coated PLGA-NPs and marketed eye drop

Ocular retention study of LFV-CS-PLGA-NPs-Opt and marketed formulation in the cul-de-sac of rabbit eye was done by gamma scintigraphy using Tc^99m ^as rediolabelling agent. The levofloxacin and NPs labelled with Tc^99m ^and labeling efficiency was checked by instant thin layer chromatography (ITLC) using acetone as mobile phase. The maximum labeling efficacy was found to be 97% in stannous chloride (50 µg) at pH 7.5. The radiolabelled LFV-CS-PLGA-NPs-Opt and marketed formulation was installed into cul-de-sac of rabbit eye and retention was observed. The LFV-CS-PLGA-NPs-Opt suspension showed significance (*P < *0.05) higher retention on cornea surface at 5 h without any significance radioactive labelled NPs in other body as compared to the pure drug solution (Figures 10A and 10B). The pure drug solution showed significant radioactivity in other body part, it means it was significantly eliminated from the ocular region and reached the systemic circulation through nasolachrymal duct (Figure 10B). It is also observed that the radioactivity count (count%) rapid fall in case of drug solution as compared to CS coated PLGA-NPs suspension at 30min of study (Figure 10C). The high retention of NPs on surface of cornea is due binding interation of positive charge of chitosan with negative charge of mucine (40) of eye as well as nanosize of particles. LFV-CS-PLGA-NPs-Opt were retained significantly longer duration on ocular surface (*P *< 0.05) than the aqueous levofloxacin solution, and it is strongly agreement with previous published research work (41). 

*Stability studies*

Many factors affects on stability of the prepared formulation during the whole manufacturing process like active and inactive interaction, effect of packaging material, manufacturing process *etc.*, therefore, from period of manufacturing of a particular formulation to its usage, stability is crucial. The stability study was conducted for six months as per ICH guidelines. The drug content of particular samples was analyzed by HPLC. It was observed that there is no significant (*P *> 0.05) changes in particle size (two tailed *P *= 0.18), encapsulation efficiency (two tailed *P *= 0.17), and drug loading (two tailed *P *= 0.178) from the zero time data. The degradation rate constant was very low (0.67 × 10^-4^, as the overall degradation is <4%). The tentative shelf life was found to be 2.2 years at 25 ºC (42).

**Table 1.** In depended and dependent variable used in statistical designing and their levels

**Factor**	**Level used, actual (coded)**		
Independent variable	Low (-1)	Medium (0)	High (+1)
A= PVA	1	2	3
B= PLGA (%)	0.2	0.4	0.6
C= Chitosan	0.2	0.6	1
**Dependent variables**			
Y_1_= Particle size	Minimize (<200 nm)		
Y_2_= Encapsulation Efficiency (EE)	Minimize		
Y_3_= Drug loading	Maximize		

**Table 2:** Actual and predicted value of all batches

**Run order**	**Formulation code**	**PVA (%)**	**PLGA (%)**	**Chitosan (%)**	**Particle size (nm)**		**Entrapment efficiency (%)**		**Drug loading (%)**	
					**Actual**	**Predicted**	**Actual**	**Predicted**	**Actual**	**Predicted**
1	F1	1.00	0.40	0.20	156.21 ± 15.23	152.16	20.54 ± 6.25	21.80	9.24 ± 2.12	9.09
2	F2	2.00	0.40	0.60	170.32 ± 13.67	169.97	49.21 ± 2.54	49.45	11.21 ± 1.43	11.29
3	F3	3.00	0.40	0.20	102.60 ± 20.54	104.81	38.31 ± 5.76	39.49	15.26 ± 1.98	15.32
4	F4	3.00	0.40	1.00	143.24 ± 16.21	147.29	59.54 ± 2.22	58.28	13.85 ± 1.54	14.00
5	F5	1.00	0.60	0.60	190.54 ± 11.86	192.92	37.43 ± 5.75	37.61	3.85 ± 1.23	3.91
6	F6	2.00	0.40	0.60	169.43 ± 15.23	169.97	49.65 ± 3.23	49.45	11.23 ± 1.54	11.29
7	F7	3.00	0.20	0.60	116.40 ± 18.21	114.02	40.23 ± 4.12	40.05	16.01 ± 1.01	15.95
8	F8	2.00	0.20	0.20	128.12 ± 17.32	128.29	26.41 ± 6.43	25.40	14.02 ± 1.21	14.01
9	F9	1.00	0.20	0.60	187.54 ± 10.43	191.42	20.43 ± 5.34	20.18	10.44 ± 2.4	10.60
10	F10	1.00	0.40	1.00	208.76 ± 8.54	206.55	38.32 ± 3.54	37.14	8.09 ± 3.2	8.19
11	F11	2.00	0.20	1.00	180.76 ± 9.76	179.10	48.54 ± 2.54	49.97	12.06 ± 2.13	11.96
12	F12	2.00	0.40	0.60	172.35 ± 14.54	169.97	49.11 ± 3.25	49.45	11.43 ± 1.43	11.29
13	F13	2.0	0.60	0.20	154.61 ± 13.86	156.27	51.32 ± 2.05	49.89	8.09 ± 3.65	8.19
14	F14	2.00	0.40	0.60	168.32 ± 14.90	169.97	49.76 ± 2.34	49.45	11.28 ± 1.38	11.29
15	F15	2.00	0.40	0.60	169.42 ± 13.51	169.97	49.54 ± 2.43	49.45	11.32 ± 2.13	11.29
16	F16	2.00	0.60	1.00	202.50 ± 9.21	202.33	58.43 ± 1.12	59.44	6.87 ± 4.23	6.88
17	F17	3.00	0.60	0.60	167.60 ± 12.54	163.72	56.32 ± 1.49	56.57	11.90 ± 2.12	11.74

**Table 3:** ANOVA of calculated model for responses

**Result of the analysis of variance**	**Particle size (nm)**	**Entrapment efficiency (%)**	**Drug loading** **(%)**
**Regression**			
Sum of squares	13029.42	2348.04	157.64
Degrees of freedom (df)	9	9	9
Mean squares	1447.71	260.89	17.52
*F*-value	102.64	144.65	754.55
*P*	<0.0001	<0.0001	<0.0001
**Lack of fit tests**			
Sum of squares	89.63	12.31	0.13
Degrees of freedom (df)	3	3	3
Mean squares	29.88	4.10	0.044
*F*-value	13.13	51.72	5.77
*P*	0.0154	0.0012	0.0618
R^2^	0.9925	0.9947	0.9990
Correlation of variation (CV%)	2.29	3.07	1.40

**Table 4:** Regression analysis of all responses (Y_1_, Y_2_ and Y_3_).

**Model**	**R** ^2^	**Adjusted R** ^2^	**Predicted R** ^2^	**SD**	**CV (%)**	**Remark**
**Response (Y**_1_ **)**						
Linear	0.8901	0.8647	0.7834	10.54	-----	-----
2F1	0.9374	0.8999	0.7349	9.06	-----	-----
Quadratic	0.9925	0.9828	0.8897	3.76	2.29	Suggested
**Response (Y**_2_ **)**						
Linear	0.8102	0.7664	0.6702	5.87	---	----
2F1	0.8355	0.7367	0.4352	6.23	-----	-----
Quadratic	0.9947	0.9878	0.9164	1.34	3.07	Suggested
**Response (Y**_3_ **)**						
Linear	0.9636	0.9552	0.9289	0.66	-----	----
2F1	0.9750	0.9600	0.8929	0.63	-----	----
Quadratic	0.9990	0.9976	0.9863	0.15	1.40	Suggested

Particle size (Y1) = +169.97 - 26.65A + 12.80B + 24.21C + 12.05AB - 2.98AC - 1.19BC - 9.12A^2^ + 4.67B^2^ - 8.14C^2^.

Entrapment efficiency (Y2) = +49.45 + 9.71A + 8.49B + 8.53C - 0.23AB + 0.86AC - 3.76BC - 8.92A^2^ - 1.93B^2^ - 1.35C^2^.

Drug loading (Y3) = +11.29 + 3.30A - 2.73B - 0.84C + 0.62AB + 0.18AC + 0.18BC + 0.18A^2^ - 0.93B^2^ - 0.11C^2^.

**Table 5.** Regression value of different kinetic models

**Model**	**Equation**	**Parameters**	
Zero order	mo−m = kt	r^2^	0.903
First order	ln m = kt	r^2^	0.941
Higuchi’s model	mo−m = kt1/2	r^2^	0.982
Korsmeyer-Peppas	log (mo−m) = log k + n log t	r^2^	0.960

## Conclusion

Levofloxacin loaded CS-coated PLGA-NPs were successfully developed by single emulsification solvent evaporation technique and optimized by 3-factors 3-levels Box-Behnken statistical design software. Optimal particle size, with <0.5 PdI and positive zeta potential value was observed with the developed nanoparticles. An extended release with prolonged retention and better tolerability at corneal site was observed as compared to marketed levofloxacin eye drop. The antimicrobial activity of developed NPs was better as compared to the marketed levofloxacin eye drop. Irritancy and gamma scintigraphy study revealed that the LFV-CS-PLGA-NPs-Opt is non irritant and prolong retention on corneal surface as well as reduced nasolachrymal drainage and safe in use. The developed LFV-CS-PLGA-NPs-Opt showed a good stability profile making it suitable for enhancing and prolonged release of drug on ocular surface and warrants clinical evaluation and application.
